# Design of Optical System for Ultra-Large Range Line-Sweep Spectral Confocal Displacement Sensor

**DOI:** 10.3390/s24030723

**Published:** 2024-01-23

**Authors:** Weiguang Yang, Jian Du, Meijie Qi, Jiayue Yan, Mohan Cheng, Zhoufeng Zhang

**Affiliations:** 1 Key Laboratory of Spectral Imaging Technology CAS, Xi’an Institute of Optics and Precision Mechanics, Chinese Academy of Sciences, Xi’an 710119, China; yangweiguang@opt.ac.cn (W.Y.); dujian@opt.ac.cn (J.D.); qimeijie0021@163.com (M.Q.); yanjiayue21@mails.ucas.ac.cn (J.Y.); chengmohan22@mails.ucas.ac.cn (M.C.); 2 University of Chinese Academy of Sciences, Beijing 100049, China

**Keywords:** spectral confocal, precision measurement, dispersive objective, optical design, imaging spectrometer

## Abstract

The spectrum confocal displacement sensor is an innovative type of photoelectric sensor. The non-contact advantages of this method include the capacity to obtain highly accurate measurements without inflicting any harm as well as the ability to determine the object’s surface contour recovery by reconstructing the measurement data. Consequently, it has been widely used in the field of three-dimensional topographic measuring. The spectral confocal displacement sensor consists of a light source, a dispersive objective, and an imaging spectrometer. The scanning mode can be categorized into point scanning and line scanning. Point scanning is inherently present when the scanning efficiency is low, resulting in a slower measurement speed. Further improvements are necessary in the research on the line-scanning type. It is crucial to expand the measurement range of existing studies to overcome the limitations encountered during the detection process. The objective of this study is to overcome the constraints of the existing line-swept spectral confocal displacement sensor’s limited measuring range and lack of theoretical foundation for the entire system. This is accomplished by suggesting an appropriate approach for creating the optical design of the dispersive objective lens in the line-swept spectral confocal displacement sensor. Additionally, prism-grating beam splitting is employed to simulate and analyze the imaging spectrometer’s back end. The combination of a prism and a grating eliminates the spectral line bending that occurs in the imaging spectrometer. The results indicate that a complete optical pathway for the line-scanning spectral confocal displacement sensor has been built, achieving an axial resolution of 0.8 μm, a scanning line length of 24 mm, and a dispersion range of 3.9 mm. This sensor significantly expands the range of measurements and fills a previously unaddressed gap in the field of analyzing the current stage of line-scanning spectral confocal displacement sensors. This is a groundbreaking achievement for both the sensor itself and the field it operates in. The line-scanning spectral confocal displacement sensor’s design addresses a previously unmet need in systematic analysis by successfully obtaining a wide measuring range. This provides systematic theoretical backing for the advancement of the sensor, which has potential applications in the industrial detection of various ranges and complicated objects.

## 1. Introduction

In industrial production processes, it is often necessary to detect the three-dimensional shape of components or products in real time in order to monitor production quantities [1,2,3]. For instance, the process of producing cell phones requires the identification of extremely small flaws, whereas the creation of chips necessitates the detection of defects on the wafer. In order to ensure the accuracy of measuring the three-dimensional shape of small parts, it is necessary to employ real-time surface detection techniques. This can be achieved through two methods: contact-based and non-contact-based measurements. Direct contact during measurement has the potential to harm the surface of the object being measured, thus compromising the accuracy of the measurement. Consequently, the development of high-precision measurement is underway as a replacement for non-contact measurement. Due to the potential for surface contamination and decreased measurement accuracy, high-precision techniques are increasingly transitioning towards non-destructive testing in order to avoid contact measurement. Most non-contact measurement techniques are primarily optical, utilizing various visual methods that have resulted in the creation of other devices [4,5,6], with spectral confocal technology being the most prominent among them.

Spectral confocal displacement sensors are being increasingly utilized in many industries, such as aerospace, microelectronics production, and biomedical domains, due to their advantageous features, including non-contact operation, small focus, high precision, and flexible integration [7,8,9,10]. They have become a powerful instrument for detecting three-dimensional morphology, indicating the emerging trend in three-dimensional morphology measuring sensors. The spectral confocal displacement sensor utilizes the axial chromatic aberration of the lens to accurately measure different wavelengths of light using the same lens. This is possible because the refractive index of the lens varies with the wavelength, causing the different wavelengths of light to focus at different positions away from the optical axis. The imaging spectrometer is then used to determine the wavelength of the light reflected from the object’s surface, establishing a relationship between the wavelength and the distance. Finally, a mechanical scanning process is employed. The acquisition of the object’s three-dimensional topographical data is accomplished [11].

The manufacturing sector has experienced rapid growth, leading to a substantial increase in both the diversity and quantity of parts and products manufactured. The conventional point-scan spectral confocal displacement sensor has limitations in its ability to estimate surface depth, as it can only do so at a single spot. Furthermore, it necessitates a very precise mechanical displacement platform for two-dimensional scanning in order to evaluate the complete three-dimensional dimensions of an item. Presently, there is a significant emphasis on point-scan displacement sensors in both local and foreign studies on confocal displacement sensors [12,13,14,15]. Researchers have suggested enhancing the spectral confocal displacement sensor to address the manufacturing industry’s need for faster measurement speeds, as the current point-scan method falls short of meeting high-speed measurement needs.

One proposed method to enhance the spectral confocal displacement sensor is by expanding the point-sweep type to the line-sweep type. STIL (France) and Precitec (Germany) have developed commercial-grade spectral confocal displacement sensors of the line-sweep kind. However, the point-sweep type remains the primary focus of research on spectral confocal displacement sensors [16,17,18]. The dispersive objective lens is the primary focus of most of the previously described point-sweep type research. Due to the widespread availability of imaging spectrometers in dispersive objective lens research, the simulation stage does not assess the complete system of the spectral confocal displacement sensor. This limitation hinders the advancement of technology in this field.

However, commercial-grade line-scanning spectral confocal displacement sensors are expensive and have a restricted measurement range. Currently, the utilization of line-scanning spectral confocal displacement sensors in production is infrequent. There is a pressing requirement for a comprehensive theoretical investigation and simulation design of the line-scan spectral confocal displacement sensor as a means of quantifying three-dimensional topography.

The two main types of line-sweep spectral confocal displacement sensors are co-axial and biaxial, and their distinctions are based on the structure of the optical channel. The biaxial design requires a completely symmetrical visual system, resulting in the necessity for a greater quantity of lenses. The overall energy loss of the optical line is further amplified due to the intricacy and challenges involved in constructing and deploying the optical channel. Concurrently, the biaxial type’s architecture has a co-axial optical channel, requiring adequate free space for precise measurements of tight space activities, such as small hole level measurement and small hole diameter detection. Currently, there is a limited number of domestic researchers engaged in the study of spectral confocal displacement sensors [19]. The objective of this article is to conduct a comprehensive theoretical analysis and simulation design of the entire line-scanning spectral confocal displacement sensor system, considering the concerns discussed earlier. Furthermore, it introduces an innovative optical design methodology for the system. The results indicate that the system has the ability to equal or exceed the performance of internationally recognized items at the commodity level, particularly in terms of the sensor’s resolution and measuring range.

## 2. Principle Analysis of Line-Sweep Spectral Confocal Displacement Sensor

**Figure 1:**
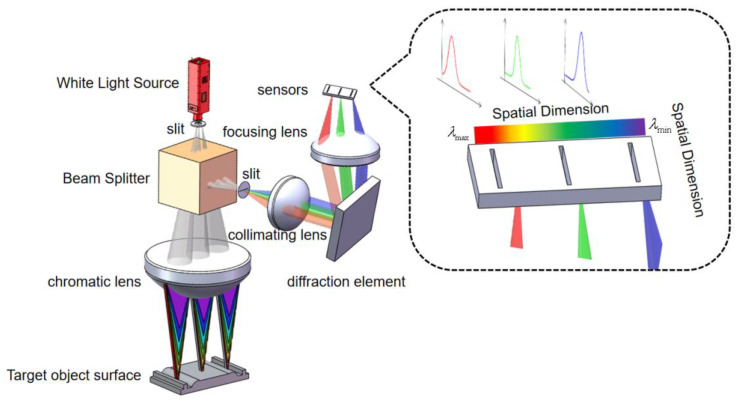
Schematic diagram of line-sweep spectral confocal displacement sensor.

The operational system of the line-sweep spectral confocal displacement sensor can be observed in Figure 1. The emitted light from the broad-band light source is enlarged into a line-shaped light source using the slit. This line-shaped light source then passes through the beam-splitter prism and the dispersive objective lens (also known as the dispersive element) to reach the surface of the object that needs to be measured. The dispersive objective lens will disperse the broadband light source into monochromatic light of varying wavelengths and focus it towards different points along the optical axis, away from the item being measured. The reflected light directed towards the object under measurement will pass via the beam-splitter prism and afterwards enter the imaging spectrometer. The object is illuminated with concentrated light, which is then reflected off the surface of the estimated object. The reflected light passes through a beam-splitter prism and enters an imaging spectrometer. Inside the spectrometer, the light is separated into different angles based on its wavelength. The peak wavelength of the energy is determined and used to establish the relationship between wavelength and displacement, also known as coding. By scanning the object in one dimension, the spectrometer obtains information about the three-dimensional topography of the object being measured.

Dispersion lenses have the ability to effectively separate light of different wavelengths, based on Figure 1. Consequently, in order to attain high-quality imaging, precise measurement outcomes, and uphold the linear relationship between the separated wavelengths, the primary factor governing the spectroscopic function of the dispersive objective lens is its inherent axial chromatic aberration. This enables the achievement of a noticeable change in color along the axis by strategically distributing axial chromatic aberration, ultimately resulting in the fulfillment of the spectroscopic function. Additionally, in order to ensure the linear relationship between the glass material qualities and the axial chromatic aberration, it is necessary to satisfy the related formula [20]:

(1) $$\left\{\begin{array}{l} \varphi =\sum_{i}^{N}\varphi_{di}\\ \sum_{i=1}^{N}\frac{\varphi_{i}\left(\lambda_{d}\right)}{\nu_{di}}\left(P_{\lambda i}-Q_{\lambda }\right)=0 \end{array}\right\}$$

In the above equation, φ denotes the optical focal length of the lens, *N* represents the number of lenses, λ represents the wavelength, $\nu_{di}=\frac{n_{di}-1}{n_{Fi}-n_{Ci}}$ denotes the Abbe number of the glass, $P_{\lambda i}=\frac{n_{\lambda i}-n_{iC}}{n_{Fi}-n_{Ci}}$ represents the relative dispersion, and $Q_{\lambda }=\frac{\sum_{i=1}^{N}\frac{\varphi_{di}}{\nu_{di}}P_{\lambda i}}{\sum_{i=1}^{N}\frac{\varphi_{di}}{\nu_{di}}}$ represents the extent of simplifying applied in the computation of the equation.

For the imaging spectrometer, the choice of its parameters depends on the parameters of the system-wide performance index. To achieve the corresponding displacement resolution ( $\delta_{\lambda }$ ), the spectral resolution ( $\delta_{d}$ ) of the spectrometer should satisfy the following formula when the dispersion range ( ΔL ) and the band range ( Δλ ) are known:

(2) $$\delta_{d}=\frac{\Delta L\times \Delta \lambda }{\delta_{\lambda }}$$

A commonly used tool in the field of research is an imaging spectrometer. It consists of various components, such as a collimator, spectroscopic elements, focusing mirrors, and detectors. Among the spectroscopic elements, prisms and gratings are the most commonly used. Prisms are used for spectroscopy based on the refractive index of the material and the angle of the top corner, while gratings rely on the logarithm of the grating line to perform spectroscopy. When compared, the grating spectroscopy offers a higher spectral resolution than prism spectroscopy, while the collimating and focusing mirrors remain unchanged. Thus, the choice of spectral resolution should be based on the specific requirements of the task at hand. Based on the necessary spectral resolution, please make a specific selection.

## 3. Design of Optical System for Ultra-Large-Range Line-Sweep Spectral Confocal Displacement Sensor

The sensor system comprises a broadband light source, a slit, a dispersive objective, a spectroscopic prism, and an imaging spectrometer. The operational bandwidth of the system is 150 nm, with a central wavelength of 575 nm. The working band covers a range from 500 nm to 650 nm. The dimensions of the detector picture are 8.5 μm by 8.5 μm, and the corresponding Nyquist frequency is 59 line pairs per millimeter (lp/mm). The optical system of the spectral confocal displacement sensor, as shown in Table 1, is designed to meet the requirements of measuring large surfaces and fast speeds.

The optical system can be divided into two main parts: the dispersive objective and the imaging spectrometer. In order to meet the performance requirements mentioned in Table 1, the allocation of indices for these two parts needs to be carefully considered. The working wavelength, scanning line length, and dispersive range of the dispersive objective are determined by the optical system based on the constraints mentioned in Equation (2) above. Once the dispersive objective design is finished, the overall system’s axial resolution is solely dependent on the spectral resolution of the imaging spectrometer.

### 3.1. Dispersive Objective Lens

The optical design methodology of the dispersive objective lens deviates from the conventional approach employed in creating optical systems. The conventional optical imaging system design will efficiently eliminate chromatic aberration. The imaging quality of the visual system is significantly affected by the secondary spectrum when high picture quality is needed. Hence, it is imperative to make adjustments for the secondary range. The dispersive objective lens does not eliminate axial chromatic aberration; rather, it deliberately enhances it by utilizing the principal aberration coefficient of the thin lens universal formula:

(3) $$S_{IC}=\sum h^{2}C$$

This amplification allows for the determination of the axial chromatic aberration during the procedure, where *h* is the projection height of the near axis leading light, $C=\sum \left(\varphi /\nu \right)$ , φ is the optical focal length of the thin lens, and ν is the dispersion coefficient (Abbe constant).

Based on the given formula, the size of the axial chromatic aberration is solely dependent on the optical focal length of the lens and the dispersion coefficient of the glass once the object field of view and the object aperture are determined. When determining the focal length at the central wavelength, it is important to keep the radius of curvature of the two sides of the lens unchanged. This ensures that the initial axial chromatic aberration of the entire system is solely dependent on the properties of the glass material. Therefore, in the design of a dispersive objective lens, the first step should be to carefully select the appropriate glass material based on the criteria mentioned in Formula (1). Equation (1) represents the criterion mentioned above.

Prior to selecting the glass material, it is imperative to consider the choice of an appropriate initial construction. The choice of the initial configuration has a significant impact on the optical design process. An efficient setup during the initial installation can greatly minimize the time and effort spent on optical design. In the past, the primary factors considered when choosing the initial configuration were the F-number and the field of view. Considering the numerous off-axis aberrations present in this design, it is crucial to carefully address aberration correction when selecting the initial configuration. Thus, it is crucial to address any abnormalities during the initial selection of the structure. Therefore, the decision was made to utilize a fully symmetrical double-Gaussian configuration in the design of the dispersive lens.

This study presents a novel approach that combines the theory of axial chromatic aberration with visual design software to develop a design for a dispersive objective optical path that effectively and accurately fulfills the design requirements. The methods in question are clearly outlined as follows:

(1) In order to address the issue of linear axial chromatic aberration, it is essential to determine the appropriate glass material. Optical design software is essential for selecting the appropriate material due to the variability of the refractive index in glass. Optical design software enables the choice of the primary glass material without compromising its optical focus. The limitations of this section are determined by Equation (1) described earlier. Upon completion of the selection process, the initial construction successfully achieves the desired level of positive axial chromatic aberration as specified in the design criteria;
(2) By maintaining a constant focal length value for the center wavelength, the optical system ensures consistent optical focal length. This helps to preserve the accuracy of correcting aberrations and maintain the integrity of the axial chromatic aberration;
(3) At the initial phase of addressing distortions, the optimization method entails making use of RMS (root mean square), primary ray, and barycentre. Once the primary aberration correction is nearly complete, the optimization strategy focuses on incorporating wavefront, primary ray, and barycentre to further correct any remaining aberrations. Glass replacement is ideal for surfaces that exhibit noticeable differences in aberration.

**Figure 2:**
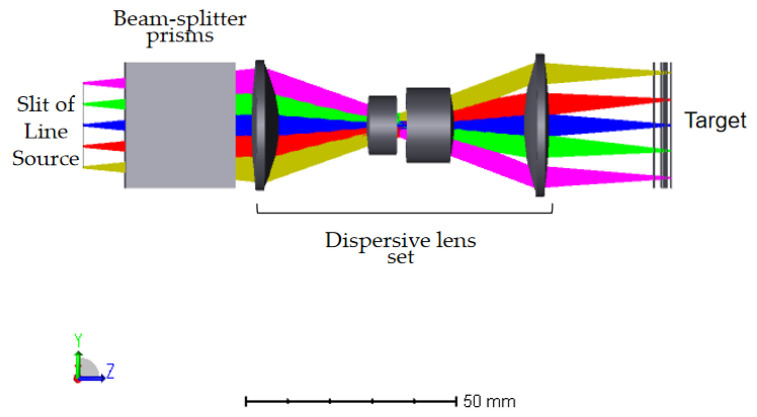
Structure of dispersive objective optical path.

Through modifications and enhancements to the initial configuration, the procedure mentioned above has resulted in the final optical pathway structure of the dispersive lens. It can be seen in the Figure 2 below. Once passing through the beam-splitter prism and dispersive objective lens set, the light emitted by the light source will be focused at different positions along the optical axis, depending on their respective wavelengths.

The optical path structure includes a dispersive objective and a beam-splitter prism to direct the reflected light into the imaging spectrometer. The dispersive objective and prisms consist of a total of seven lenses. Initially, the structure was a double Gaussian structure, which is fully symmetric and eliminates vertical chromatic aberration. However, the addition of prisms disrupts the overall symmetry, resulting in a final magnification of −1.2. This adjustment is necessary to ensure a uniform optical path for the light entering the spectrometer. To ensure uniformity of light in the spectrometer, a double telecentric optical path is employed, with the object side being telecentric. The image side necessitates the electricity to be below 0.5°, and the parameters of the final optical system can be found in Table 2 below.

### 3.2. Dispersive Objective Dispersion Range and Imaging Quality Analysis

**Figure 3:**
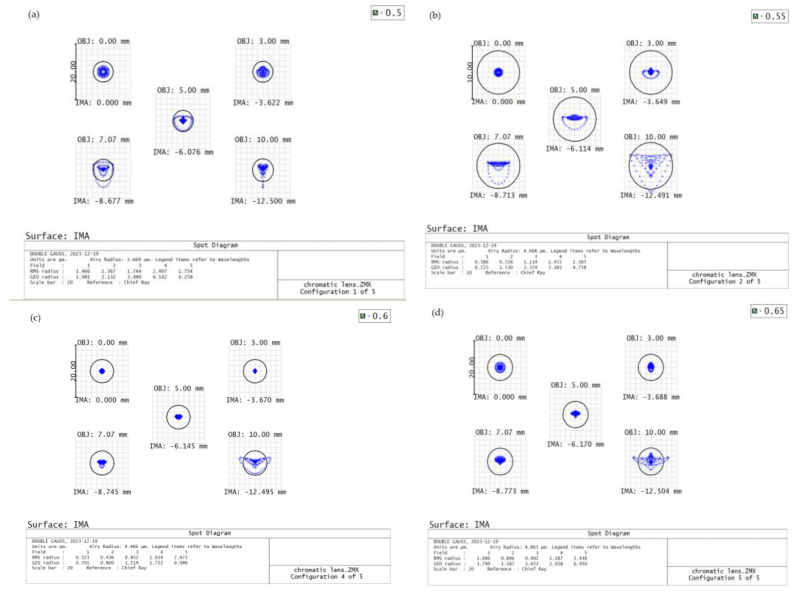
Dispersive objective (**a**) 500 nm dot plot, (**b**) 550 nm dot plot, (**c**) 600 nm dot plot, (**d**) 650 nm dot plot.

Once the optical path design of the dispersive objective lens is finished, it is important to analyze the imaging using evaluation criteria based on the spot diagram. This diagram represents the energy concentration of the optical system, and the distribution of points in the diagram can approximate the energy distribution of the image. By measuring the density of these points, we can determine the quality of the imaging, whether it is excellent or poor. It is important to analyze the imaging quality of different bands when using the dispersive objective, as it utilizes a broadband light source with a range of 500 nm–650 nm. In Figure 3 below, it can be observed that the dispersive spot diameter in the four sampling bands (500 nm, 550 nm, 600 nm, and 650 nm) within different fields of view is smaller than the image element size of 8.5 μm. This suggests that the imaging quality is of high standard.

**Figure 4:**
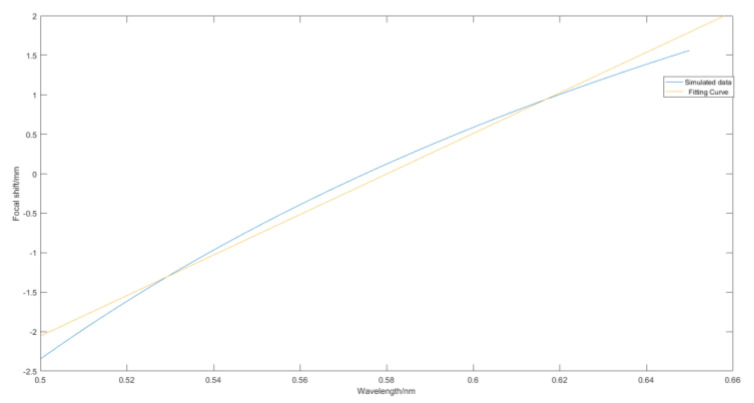
Wavelength−displacement fit.

A linear regression analysis was performed to examine the relationship between wavelength and focal shift of the dispersive lens, after simulating the data. The results of the fit are presented in the Figure 4 below. The objective demonstrates an axial chromatic aberration of 3.909 mm for the wavelength range from 500 nm to 650 nm. In addition, the value of the linear coefficient of determination *R*^2^ is determined to be 0.9944.

### 3.3. Imaging Spectrometer

This paper takes the design of the whole line-scan spectral confocal displacement sensor system as the research objective. The imaging spectrometer as the back end of the line-scan spectral confocal displacement sensor is often neglected by researchers, and buying the commercial-grade imaging spectrometer is time-saving. However, their research lacks an analysis of the whole sensor system. The primary role of the imaging spectrometer is to obtain the wavelength of the light focused on the surface to be measured to establish the mapping relationship between the wavelength and displacement; the leading indicators of the design of the imaging spectrometer are the spectral resolution and the amount of spectral bending. The spectral resolution determines the axial resolution of the line-sweeping spectral confocal displacement sensors, and the amount of spectral bending determines the accuracy of the mapping between the wavelength and the amount of displacement.

The resolution of the system is mainly determined by the focal lengths of the collimating and focusing mirrors and the spectroscopic elements. The spectroscopic elements of the imaging spectrometer are mainly prisms and gratings. Compared with prisms, gratings have a high spectral resolution, a wide spectral range, and a uniform dispersion.

**Figure 5:**
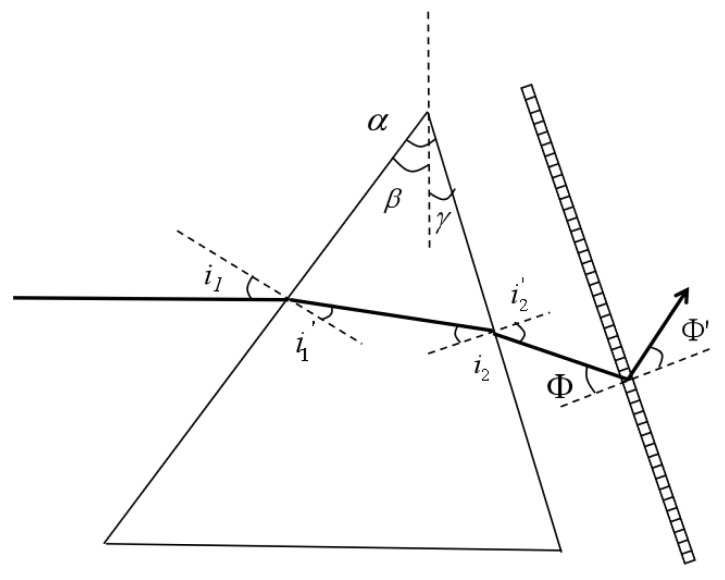
Prism grating-type spectrometer schematic.

Choosing either a grating or a prism as the spectroscopic component will cause the spectrum picture to be curved on the surface of the detector. Spectral bending occurs predominantly when light is produced in the non-primary cross-section of a prism or grating, leading to the dispersion phenomena. The spectral bending of the grating bends to the long-wave direction, and the spectral bending of the prism bends to the short-wave direction. The spectral bending characteristics of the two also give rise to the possibility of combining the two to eliminate the spectral bending of the image plane. The Figure 5 below depicts a spectrometer with a prism and a grating. The angles at which light enters the prism are referred to as the angles of incidence, labeled as i_1_ and i_2_. The angles at which light exits the prism are referred to as the angles of emission, labeled as i_1_′ and i_2_′. The increase in the occurrence of the grating is denoted by Φ, whereas the expansion of the diffraction of the grating is denoted by Φ’. The frequency of grinding has escalated, accompanied by a commensurate deflection angle referred to as Φ′. The highest point of the prism is represented by α, the angle of the first face is β (considered unfavorable in the diagram), and the angle of the second face is γ (considered positive in the figure). The grating is aligned in parallel with the second face of the prism, with an angle of incidence of Φ = i_2_′.

In order to facilitate the correction of aberration, imaging spectrometer collimator, and focusing mirror using the same focal length, the structure is an entirely symmetrical form at this time, to correct the spectral line bending, we need to ensure that the following formula is established [21]:

(4) $$\begin{array}{l} \Delta y^{\prime }=\frac{k\lambda_{0}x^{2}}{2df_{2}^{\prime }\cos\Phi }-\frac{\left({n^{2}}_{\lambda_{0}}-1\right)\sin\left(\gamma -\beta \right)}{2n_{\lambda_{0}}f_{2}^{\prime }\cos i_{1}^{\prime }\cos i_{2}^{\prime }}x^{2}=0\\ \Rightarrow \frac{\lambda_{0}}{d\cos\Phi }=\frac{\left({n_{\lambda_{0}}}^{2}-1\right)\sin\left(\gamma -\beta \right)}{n_{\lambda_{0}}\cos i_{1}^{\prime }\cos i_{2}^{\prime }} \end{array}$$

where *d* is the number of lines of the grating, *λ* is the center wavelength of the spectrometer design, *x* is the width of the slit, *k* is the diffraction level of the grating, and *n* represents the refractive index of the prism at the center wavelength. The angles of inclination *β* and *γ* of the prism’s two faces can be found by solving the preceding equation.

The grating determines the number of line pairs as 1200 line pairs/mm, the imaging spectrometer focusing mirror selects a double Gaussian structure as the initial structure. The magnification is set at 1:1, and the size of the CCD image element is 8.5 μm, which is combined with two image elements in the process of practical application. The Table 3 below displays the imaging spectrometer’s primary parameters.

**Figure 6:**
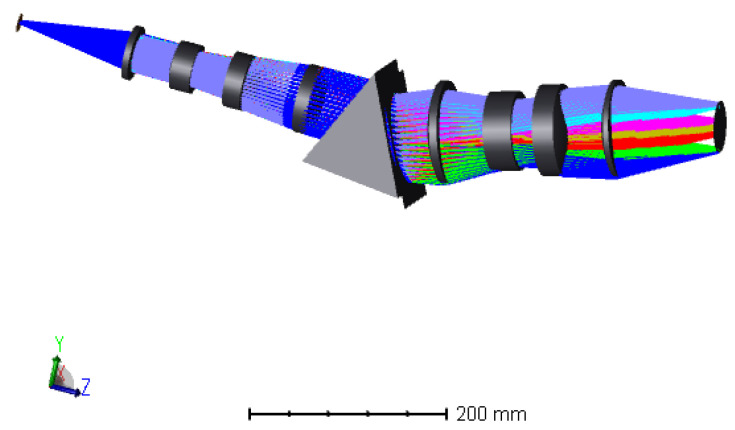
Imaging spectrometer optical path diagram.

The prism is made of H-K9L glass, which is sourced from Chengdu Guangming Company (Chengdu, China). The prism has a center wavelength of 575 nm. The tilt angle of both sides of the prism is determined through numerical computation of Equation (4), yielding the following approximate values: *β* = −26.75°, and *γ* = 23.1°. By utilizing the grating dispersion equation and considering the spectral resolution requirements of the system, the dispersion angle at the central wavelength is determined to be 7.32°. The collimating and focusing mirrors have a focal length of 230 mm. To address the challenges posed by the aperture of the dispersive objective lens and the complexity of aberration correction, an initial structure consisting of a double Gaussian configuration is chosen for the collimating and focusing mirrors. By incorporating the replacement of glass, optimizing aberration, and introducing relevant spectral bending parameters, the design specifications of the imaging spectrometer are successfully fulfilled. The design specifications of the optimized imaging spectrometer have been fulfilled. The Figure 6 below illustrates the optimized optical route.

To confirm if the imaging spectrometer’s spectral resolution aligns with the specified design criteria of 0.03 nm, it is essential to acquire the spectral response function curves for each band of the spectrometer. However, the optical design software does not provide a direct measurement of the spectral resolution value. Therefore, the spectrometer’s data are extracted using the formula outlined below. The simulation program is used to simulate the spectral response function curves.

(5) $$SFR\left(y\right)=\left[rect\left(\frac{y}{a}\right)\times LSF_{T}\left(y\right)\right]\times rect\left(\frac{y}{a}\right)$$

where *a* represents the width of the slit, *SFR* represents the spectral response function curve, the convolution is represented by “ × “, and the rectangular functions are represented by “*rect*”, with the former representing the incident slit function and the latter representing the detector image element response function. The variable *y* represents the coordinates of the image surface in the midday direction, and *LSF_T_* represents the *y* position at the line diffusion function. By integrating the diffusion function along the *y* coordinates of the slit length direction, we obtain the corresponding *y* position [22].

**Figure 7:**
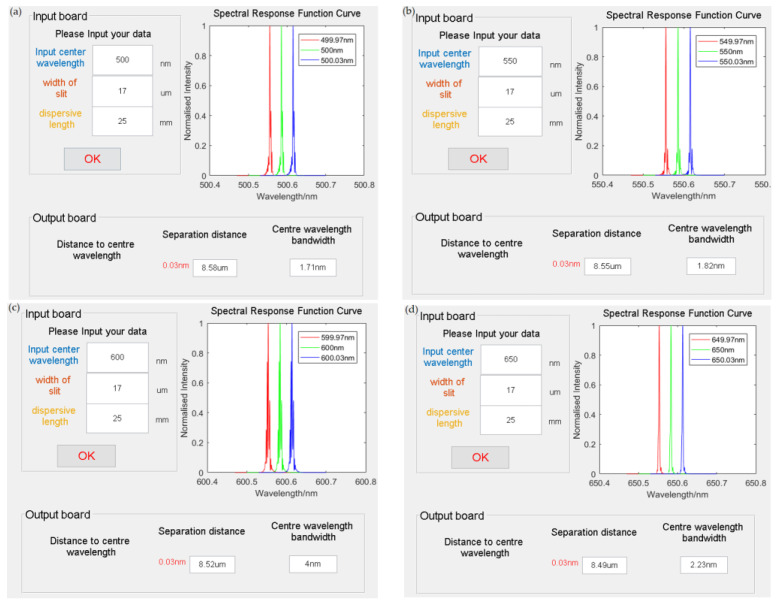
Spectrometer (**a**) 500 nm spectral response function curve simulation, (**b**) 550 nm spectral response function curve simulation, (**c**) 600 nm spectral response function curve simulation, (**d**) 650 nm spectral response function curve simulation.

The design requirements state that the imaging spectrometer must be able to resolve wavelengths as small as 0.03 nm. The Figure 7 below illustrates the spectral response curve obtained by inputting the necessary data (center wavelength, slit width, dispersion width) into the input panel. The input center wavelength interval is set to 0.03 nm. The output panel confirms that the wavelengths, spaced 0.03 nm apart, are more significant than the size of an image element (8.5 μm). This demonstrates that the converted spectrometer has a resolution of 0.03 nm.

**Figure 8:**
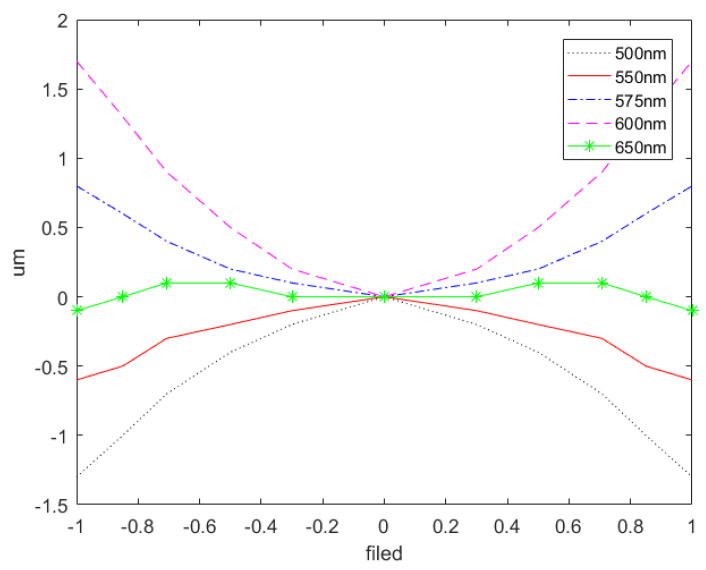
Plot of spectral line-bending results of PG type imaging spectrometer.

During the design process of an imaging spectrometer, spectral bending occurs. When the magnification is 1:1, the amount of spectral bending is not affected by the focal length of the objective lens. Instead, it is directly proportional to the emission angle of the non-central wavelength and the square of the difference between the emission angle and the central wavelength. As the length of the slit increases, the amount of spectral bending also increases. Consequently, the spectral lines are bent towards the direction of the wider field of vision. By optimizing the central wavelength spectral bending, the prism-grating imaging spectrometer is designed to achieve symmetric distribution of the remaining spectral bending after completing the correction of central wavelength spectral bending. The Figure 8 below illustrates the spectral line bending of four selected bands: 500 nm, 575 nm, 600 nm, and 650 nm. It is evident that the spectral line bending does not exceed 2 μm, thus satisfying the design criteria.

### 3.4. Imaging Quality Analysis

Upon finishing the optical design of the imaging spectrometer, it is imperative to assess the imaging quality. In this case, the point spread diagram and the modulation transfer function (MTF) curve are utilized as the means of evaluating the imaging quality of the imaging spectrometer. The modulation function curve represents the optical system’s ability to transmit different frequencies to various objects. It is the most comprehensive approach for evaluating this ability.

**Figure 9:**
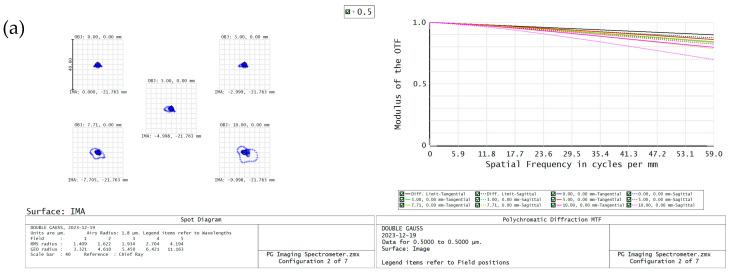

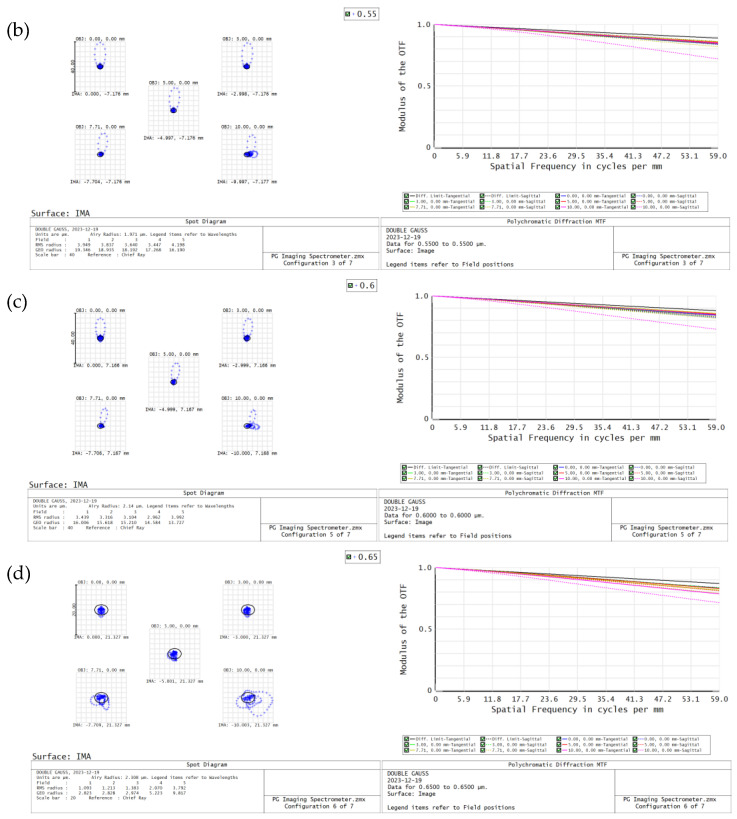
Imaging spectrometer (**a**) 500 nm spot list with MTF curve, (**b**) 550 nm spot list with MTF curve, (**c**) 600 nm spot list with MTF curve, (**d**) 650 nm spot list with MTF curve.

Four sample bands, specifically at wavelengths of 500 nm, 550 nm, 600 nm, and 650 nm, were chosen to assess the image quality across the whole field of view. The MTF modulation function curves were incorporated as the standardized measure for evaluating the imaging quality. The Figure 9 below demonstrates that the imaging spectrometer has a diffuse spot diameter of less than 8.5 μm across the full field of view. Additionally, the Nyquist cut-off frequency of 59 lp/mm exceeds 0.5, indicating excellent imaging quality that fulfills the design specifications.

## 4. System-Wide Optical Path Analysis

**Figure 10:**
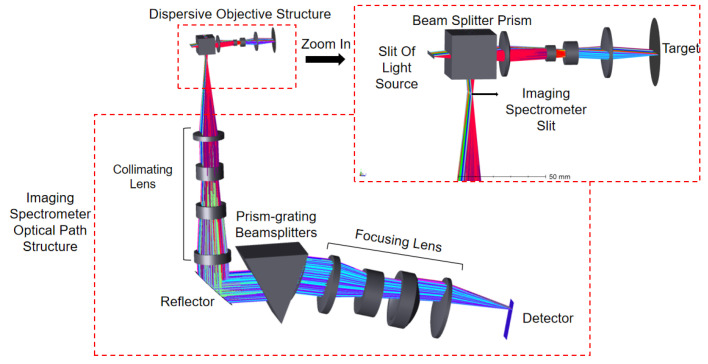
Synthesized optical path diagram of the whole system.

Once the optical design of the dispersive objective and imaging spectrometer is finished, along with the analysis of imaging quality, it becomes essential to evaluate the imaging quality of the entire system. The optical structure of the system is constructed based on the principle of the line-sweeping spectral confocal displacement sensor. To simulate the light reflected from the measured object, the object being measured is set up as a specular reflection. To reduce the size of the optical route in the imaging spectrometer, mirrors are incorporated to fold the path. The configuration of the entire optical path system is illustrated in Figure 10.

**Figure 11:**
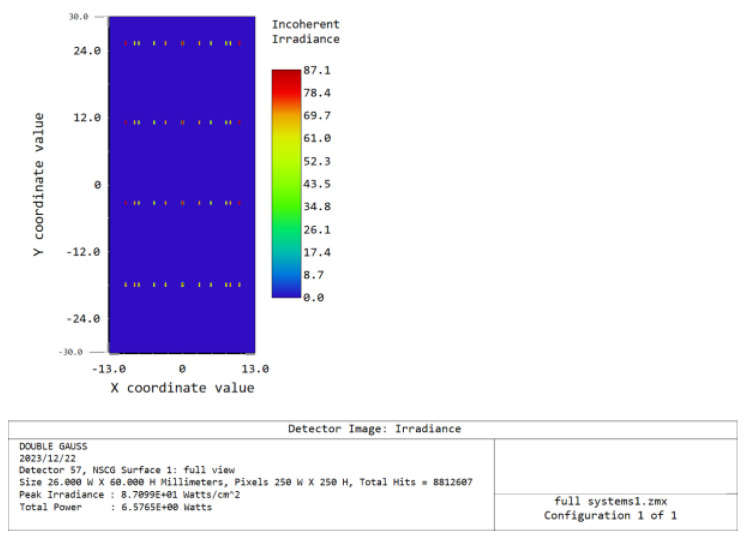
System−wide ray−tracing light path diagram.

Once the optical route construction is finished, it is essential to trace the light across the entire system in order to receive the imaging results. The light tracing was conducted with center wavelengths of 500 nm, 550 nm, 600 nm, and 650 nm. The detector of the imaging spectrometer captures four wavelengths of light: 500 nm, 550 nm, 600 nm, and 650 nm. These wavelengths are displayed in Figure 11, arranged from top to bottom.

The radius of the diffuse spot in the entire field of view of the ray tracing is indicated in Table 4. Subsequently, the method of merging two pixels is employed during the practical application. This can be observed as the radius of the scattered area in the ray tracing of the entire system is lower than the dimensions of a single pixel, thereby aligning with the established design specifications.

Upon completion of the system analysis, performance indexes of the dispersion lens designed in this chapter and the STIL (MPLS-DM SUPERVIEW) line-sweeping spectral confocal displacement sensor, a leading foreign product, are compared in Table 5. The data in Table 5 clearly demonstrate that the dispersion lens designed in this chapter outperforms the STIL products in terms of scanning line length, measuring range, axial resolution, and working distance.

## 5. Tolerance Analysis

Once the design of an optical system is finished, it is essential to conduct a tolerance analysis to assess the system’s practicality in terms of processing. The purpose of tolerance analysis for optical and manufacturing engineers is to verify that the bridge meets the system’s performance requirements by defining and distributing tolerances in a fair manner. This helps minimize costs and assure the best quality in the manufacturing of the finished product. The lack of tolerance results in higher processing costs. It is possible that there are increasing issues; thus, an optical system can possess exceptional imaging quality and satisfy the contemporary processing standard of tolerance [23].

**Figure 12:**
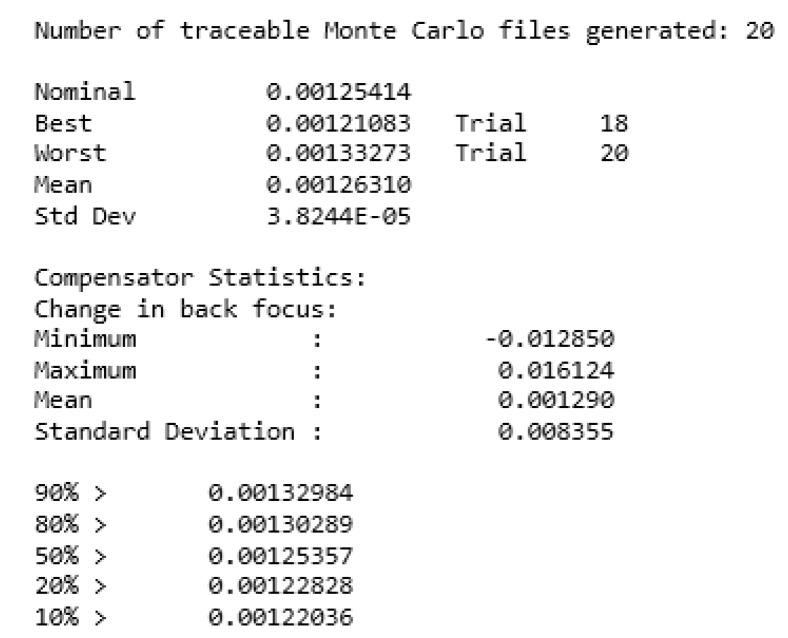
Monte Carlo analysis of dispersive objectives.

The optical design software is utilized to assess the tolerance of both the dispersive objective lens and the imaging spectrometer. The RMS spot radius is employed as the sensitivity for the dispersive objective lens, with the actual machining level determining the set tolerance: the lens surface has a radius of curvature of ±0.02 mm. Each surface has a thickness of ±0.05 mm, an eccentricity of ±0.03 mm, and a tilt of ±0.05°. The element tilt tolerance is ±0.03 mm and ±0.05°. The element eccentricity tolerance is ±0.03 mm. The Monte Carlo analysis results are depicted in the accompanying figure. The Figure 12 below demonstrates that the tolerance established by the actual processing level satisfies the imaging quality criteria of the dispersive objective lens.

**Figure 13:**
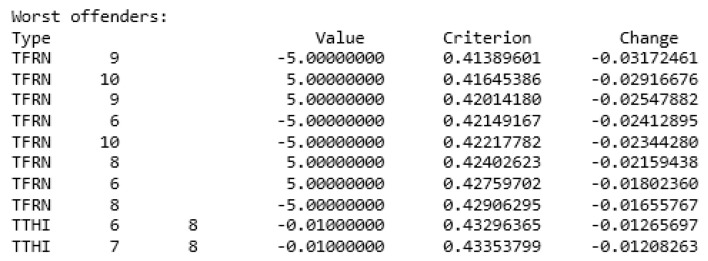
Imaging spectrometer tolerance analysis results.

The modulation transfer function (MTF) curve of the imaging spectrometer is determined by the tolerance sensitivity and the actual level of machining used to set the tolerance: a radius of curvature of each lens surface tolerance of 5 fringes, a thickness of each surface tolerance of ±0.01 mm, a surface eccentricity tolerance of ±0.01 mm, a surface tilt tolerance of ±0.02°, an element eccentricity tolerance of ±0.01 mm, and a component tilt tolerance of ±0.01 mm. Element tilt tolerance is ±0.03°. The Figure 13 below displays the results of the tolerance analysis.

**Figure 14:**
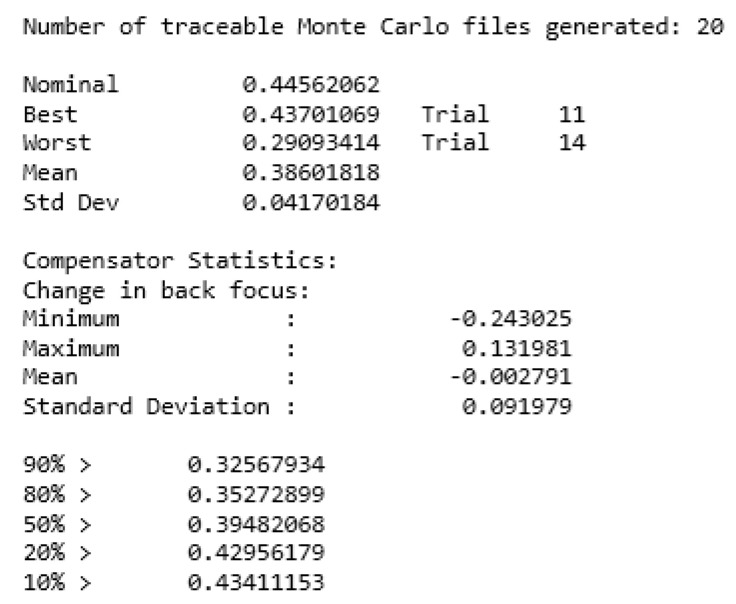
Imaging spectrometer Monte Carlo analysis chart.

The outcomes of the Monte Carlo analysis are depicted in the Figure 14 below. According to the findings of the Monte Carlo analysis, the components that are sensitive to tolerance are carefully regulated during the machining or assembly process to prevent any negative effects on the entire system.

## 6. Discussion

This paper focuses on the design of the line-scan spectral confocal displacement sensor. It proposes an optical design method based on chromatic aberration theory and optical design software. This method is suitable for designing the line-scan dispersive objective lens. It addresses the issue of blindness in the current optical design process and reduces the difficulty of designing the dispersive objective lens for the sensor. Additionally, it includes the design of the back-end imaging. Simultaneously, the development of the back-end imaging spectrometer was undertaken, along with an analysis of the optical structure and imaging quality of the entire system. This analysis provided a strong theoretical foundation for the advancement of the principle prototype of the line-scanning spectral confocal displacement sensor, expediting the research progress of said sensor. The paper presents a line-scanning spectral confocal displacement sensor system with a scanning line length of 24 mm, a measuring range of 3.9 mm, and a theoretical axial resolution of 0.8 μm. This demonstrates the superiority of the proposed optical design method over the existing products of STIL. In the future, there is an expectation to enhance the design method in order to achieve a wider measuring range. Additionally, there will be efforts to conduct engineering research on line-scanning spectral confocal displacement sensors and develop a new model of this type of sensor. Simultaneously, the engineering investigation of the line-sweep spectral confocal displacement sensor will be conducted, and the sensor will be utilized in diverse domains to perform precise measurements of distinct displacements.

## Figures and Tables

**Table 1:** Line-sweep spectral confocal displacement sensor performance specifications.

Parameters	Value
Wavelength range	500–650 nm
Scanning line length	≥20 mm
Dispersion range	≥3 mm
Axial resolution	≤0.8 μm

**Table 2:** Dispersive objective optical system parameters.

Parameters	Value
Center wavelength focal length	120 mm
Magnification	−1.2
Scanning line length	24 mm
Objective field of view	10 mm
Working distance	25 mm
Aperture of object	0.1

**Table 3:** Parameters of the imaging spectrometer.

Parameters	Value
Spectral range	490–660 nm
Aperture of object	0.15
Spectral resolution	0.03 nm
Spatial resolution	8.5 μm
Spectral line bending	Smile < 2 μm Keystone < 2 μm
Slit Size	10 mm × 17 μm

**Table 4:** Dispersive spot sizes at sampled wavelengths for system-wide ray tracing.

Wave band	500 nm	550 nm	600 nm	650 nm
Diffuse spot radius	6.724 μm	6.715 μm	6.693 μm	6.656 μm

**Table 5:** Performance parameter comparison.

Researches	Scanning Line Length	Measurement Range	Axial Resolution	Working Distance
This paper	24 mm	3.9 mm	0.8 μm	25 mm
STIL Corporation	12.85 mm	2.6 mm	1.8 μm	16 mm

## Data Availability

No new data were created.
